# Preventative Intervention for Social, Emotional and Behavioural Difficulties in Toddlers and Their Families: A Pilot Study

**DOI:** 10.3390/ijerph16040569

**Published:** 2019-02-16

**Authors:** John McAloon, Karina D. Lazarou

**Affiliations:** Discipline of Clinical Psychology, Graduate School of Health, University of Technology Sydney, Sydney, NSW 2007, Australia; Karina.D.Lazarou@alumni.uts.edu.au

**Keywords:** prevention, early intervention, toddlers, child behaviour, emotional problems, parenting

## Abstract

Preventative intervention early in life is key to interrupting trajectories toward subsequent emotional and behavioural problems later in life. This study examined the effectiveness of the Holding Hands program, an innovative program of preventative intervention aimed at improving the behavioural and emotional functioning of 12 to 48-month-old toddlers, and the wellbeing of their parents. This program seeks to synthesise the existing evidence in four ways; it incorporates both traditional Parent Management Training and Direct Coaching methods. It is intensive, significantly reducing session numbers and it explicitly addresses parental emotion regulation. The program also utilises operant learning principals in an effort to contingently reinforce behaviour that parents want to see more of, without employing exclusionary strategies in response to behavior that parents want to see less of. Thirty-one families, with a toddler who met clinical or sub-clinical cut-offs for externalising or internalising problems, were self- or externally-referred to the six- to eight-week program. Results indicated statistically significant improvement in toddler emotional and behavioural functioning, and parent well-being on a range of psychometric measures from pre- to post-treatment. Treatment gains were maintained for parents and children at follow-up. Implications of these findings for clinical practice and suggestions for future research are discussed.

## 1. Introduction

Emotional and behavioural problems in young children represent a common reason for referral to psychological services [1,2]. These problems are relatively stable without treatment, and may persist into adolescence and adulthood in the form of internalising disorders, such as anxiety or depression, or as externalising disorders such as Oppositional Defiant Disorder and Conduct Disorder [2,3,4,5]. Emotional and/or behavioural difficulties in toddlerhood have been linked to impaired functioning later in life in areas, such as academic performance, peer relationships, family functioning, physical and mental health, and employment [6,7,8,9,10,11,12,13,14,15,16,17] The treatment of emotional or behavioural problems is more difficult, and less successful, later in childhood and adulthood, and thus preventative interventions are crucial in preventing adverse outcomes for children [18,19].

Parents or caregivers of children with emotional and behavioural problems are also at increased risk of various undesirable outcomes such as elevated parenting stress [19,20,21,22], decreased parenting confidence [23,24], emotional difficulties [25,26,27,28] and poor physical health [29]. Parenting stress may adversely affect parents’ behaviours, thereby increasing the risk of less responsive and more authoritarian parenting practices [30,31,32] and child maltreatment and abuse [33,34]. High parenting stress may lead to the exacerbation of child difficulties, and vice versa, creating a vicious cycle [35,36]. High parenting stress may increase attention and irritability toward difficult behaviour and the likelihood of parents initiating or engaging in coercive interchanges with their children, factors known to detrimentally impact child behaviour [37,38,39]. Parents with high levels of stress may also be more likely to model poor emotional and behavioural regulation, which may then be learnt and expressed by their children [35,40]. Interventions, which seek to improve child emotional and behavioural functioning, may thus also target parent well-being in order to maximise treatment outcomes for families [41].

Behavioural Parenting Interventions (BPIs) have been widely adopted as the most effective form of early intervention for emotional and behavioural problems [19,42]. This makes sense, given the pivotal role that parents play in their children’s developing skills [43], and the limited capacity for very young children to engage in treatment. BPIs draw on attachment [44,45] and social learning theories [46,47], as well as operant conditioning theory [48] and coercion theory [36,49,50]. According to attachment theory [44,45], children whose parents respond to their behaviour warmly and sensitively feel greater security in their relationships, and develop better emotional and behavioural skills, than parents who are harsh, controlling, or rejecting [51,52,53,54,55,56]. Social learning theory [46,47] states that children learn through modelling, observation, and reinforcement, and thus parents who exhibit or reinforce maladaptive behaviours may inadvertently contribute to the development or maintenance of problems in their children [57,58,59]. Operant conditioning theory posits a reciprocal relationship between an individual’s behaviour and the consequences of that behaviour, that is, the manner in which the behaviour operates on the environment [48]. Finally, according to coercion theory [36,49,50], difficult behaviour is established and maintained by a cycle of inadvertent reinforcement of undesirable behaviour. More specifically, undesirable child behaviour is actively engaged by parents, commonly in an effort to suppress it, thereby establishing a relational style that is inherently coercive and, in the process, potentially contingently and positively reinforcing it. The implication is that parents learn that, if effective, their parental strategies must necessarily be coercive and, if unsuccessful, that they are ineffective as parents. By contrast, children learn that in order to prevail in interpersonal interactions, they must respond with coercive responses and, by extension, that interpersonal relationships are inherently coercive. Collectively, these findings inform the theory and content of BPIs.

BPIs such as Parent Management Training (PMT) [58,60] and Parent-Child Interaction Therapy (PCIT) [61] pursue emotional and behavioural improvements by targeting parenting style and parental response to child behaviour. Parenting styles and behaviours that increase the risk of child emotional and behavioural problems include modelling and inadvertent reinforcement of maladaptive behaviours [57,62], high levels of parental control (i.e., limiting child autonomy and independence), and parental rejection (low levels of warmth, approval, and responsivity), which are each addressed in treatment [52,53,54,56]. BPIs encourage parental modelling of appropriate emotional and behavioural skills (e.g., persisting when frustrated, being brave when anxious) and contingent reinforcement of desirable child behaviour through praise, attention, and rewards [58,59,60]. Reinforcement is removed from undesirable behaviours through selective ignoring, and more severe behaviours are addressed through the withdrawal of reinforcers or exclusionary strategies [58]. Increased parental warmth, approval, and sensitivity is achieved in BPI’s through encouraging regular one-on-one time, in which parents allow their child to lead play activities while they respond through description, praise, and imitation [57]. This not only improves the quality of the parent-child relationship and encourages parents to be attuned to their child’s needs [17,60], but may also increase children’s perceived mastery and control of the home environment [63]. BPIs facilitate appropriate levels of control and autonomy in the home, as parents are taught to set firm boundaries and to provide effective commands, whilst refraining from coercive or disproportionate discipline. While it is essential that parents remain calm and non-reactive in difficult interactions with their children [2,41,60], no programs were identified that explicitly targeted parent’s ability to manage their emotions in this context.

### 1.1. The Delivery of Behavioural Parent Training Interventions

BPIs are typically delivered in two distinct modes, including the delivery of parenting information to parents in the absence of their children (i.e., PMT models) or through coaching parents as they interact with their child in session (i.e., Direct Coaching models). In PMT models, skills are delivered to parents through didactic instruction, modelling of techniques by the clinician, and role play [58]. The assessment of treatment progress is typically undertaken through parental self-report and clinician administered psychometric assessment. In contrast, in Direct Coaching models such as PCIT, clinicians coach parents as they interact with their child from behind a two-way mirror [18]. The assessment of treatment progress is undertaken through parental self-reporting and clinician observation of parent-child interactions. 

Not surprisingly, evidence suggests that Direct Coaching and PMT modes of intervention each have benefits and limitations. Direct Coaching allows clinicians to provide guidance and feedback, and to model responses in real-time [18]. A meta-analysis of 77 studies reported that programs, which required parents to practice skills with their children in sessions, reported significantly larger effect sizes in terms of improved parenting skills and/or child behaviour, than programs that did not, however the relative benefit of skills practice was not quantified in the review [64]. One explanation for this benefit is that the therapist can provide immediate corrective feedback and reinforcement to ensure parents master the requisite skills [65]. There is, however, potential for bias to exist in both models. Direct observation allows clinicians to obtain first-hand information about parent-child interactions, at least as they occur in the treatment setting. In contrast, PMT models, which rely heavily on parental self-report, may limit the clinician’s ability to accurately engage characteristics of treatment response [66,67]. 

Direct Coaching modes of treatment are, however, subject to limitations, as they provide relatively little time for explanation of concepts and the rationale for techniques outside the treatment setting, which may reduce client understanding and “buy-in” to the treatment approach [58]. This may be problematic, particularly as misperceptions of treatment (e.g., of content as irrelevant, not applicable, or too demanding), low expectations of treatment efficacy, and disagreement with the treatment approach, have been linked to reduced engagement in treatment, reduced treatment attendance, and increased attrition rates [68,69]. Further, relative to PMT models, Direct Coaching models allow less time for clinicians to build rapport with parents, and a poor therapeutic alliance is associated with decreased treatment response and increased drop-out rates [69,70]. Unfortunately, the relative effectiveness of Direct Coaching models and PMT models is unknown, with efforts to compare different BPIs yielding limited insight [71]. No previous intervention was identified that sought to explicitly combine these two modes of parent training, which may contribute to addressing the limitations previously identified in each approach, thus maximising treatment outcomes for families. 

### 1.2. The Effectiveness of Behavioural Parent Training Interventions

Various clinical trials demonstrate that BPIs are effective in improving early emotional and behavioural functioning, as well as parent outcomes such as parenting stress, anxiety, depression, and parenting confidence [63,67,72,73,74,75,76,77,78,79,80,81,82,83]. Improvements have been demonstrated across both home and school contexts, and in community settings, with treatment gains maintained at follow up [66,84,85,86]. As BPIs do not directly target parent psychological functioning, improvements in this domain may result from decreased problematic child behaviours and increased positive parent-child interactions [35]. Increased parenting confidence and perceived ability to cope with difficult behaviour, achieved through acquiring effective parenting strategies, may also account for such improvements [74,87]. 

While there is a plethora of evidence supporting the use of BPIs in the treatment of early emotional and behavioural problems, limitations must be noted. A sizeable proportion of children still experience clinically significant behavioural problems following treatment [19], and attrition rates remain in the order of 40 to 60% [88]. Further, treatment effects are not always replicated, and variability across studies exists in terms of treatment effectiveness and the extent to which gains are maintained following intervention [67,75,81,82]. The effect of BPIs on internalising symptoms is less established than that of externalising symptoms, and improvements have not been clinically significant in all studies [79]. BPIs have additionally been criticised for their lack of emphasis on parent psychological functioning and self-regulation skills, despite the role of parenting stress and reactivity in exacerbating child difficulties [35,41,89]. It is unknown whether directly targeting parent emotion regulation would maximise treatment outcomes for both parents and children.

### 1.3. The Present Study

The present study seeks to trial the effectiveness of the Holding Hands Program, a behaviourally-based, preventive program of intervention, that targets change in toddlers, their parents, and characteristics of the family system they inhabit. Holding Hands synthesises a cluster of treatment components and modes of treatment delivery in a unique way. Firstly, Holding Hands is unique in that it has been designed as a program of preventative intervention for children aged 12 to 48 months. It is primarily concerned with engaging families with young children where early markers for subsequent emotional and behavioural difficulties are present. These may include, child characteristics, for instance children in the sub-clinical or clinical range for internalising or externalising difficulties, or evidence of limitations in parenting skills sufficient to inadvertently nurture current emotional or behavioural difficulties in children.

Secondly, Holding Hands relies on the body of theory and evidence amassed within social learning theory [46,47], attachment theory [44,45], operant conditioning [48], family systems theory [90,91] and coercive family process [36,49,50] for much of its content. However, it synthesises this knowledge for delivery within two distinct modes of treatment. The program provides 90-minute sessions, the first 45 min are characterised by parent(s)-therapist manualised treatment, and the second 45 min are characterised by Direct Coaching of parent(s) and the target child [61]. Given the benefits and limitations of each mode of delivery previously mentioned, a decision was taken early in the development of Holding Hands to explicitly incorporate both into the program in an effort to impart and rehearse state of the art parenting treatment components as effectively as possible.

Thirdly, Holding Hands departs from traditional PMT approaches to addressing behaviour parents want to see less of. Instead of utilising strategies, such as planned withdrawal of reinforcers, planned consequences, and exclusionary strategies, Holding Hands takes the approach that both the family system, and the characteristics that parents want to see more of in their children can be crafted solely on the basis of contingent reinforcement of desirable behaviour. Evidence is clear that, for the vast majority of typically developing children aged between 12 and 48 months, parental attention and engagement remains a most fundamental influence in their development [92,93]. If utilised strategically, and on the basis of an ability to identify characteristics they wish to see evident in their children, currently and as they develop, parental attention is anticipated to have sufficient power to change child emotional and behavioural characteristics, without teaching exclusionary strategies that are contingent on behaviour parents want to see less of. In addition, it is anticipated that parental attention has sufficient power to craft relational characteristics between referred parents and their children, when addressed in the context of preventative intervention with families of young children. For this reason, the fundamental orientation of Holding Hands is parental reinforcement, which is contingent on behavioural and emotional responses that parents want to see more of. 

Fourthly, in an effort to respond to limitations in program effectiveness, that result from parents having difficulty regulating their own emotions when responding to their children [41,90]. Holding Hands incorporates a training component explicitly directed at assisting parents to gain these skills. This involves imparting a behaviourally based strategy directed at assisting parents to reallocate their attention away from specific child behaviour that is concerning them. Reallocation of attention has been effective in improving emotion regulation in previous work [94].

Finally, Holding Hands acknowledges that, parents experiencing distress as a result of their toddlers’ behaviour, want strategies that are effective, work quickly, and fit their families. In seeking to deliver this outcome, Holding Hands typically provides only six to eight sessions per family, and builds on the immediate delivery of systemic, behavioure-based strategies with specific problem-solving tasks and relational enhancement. It has been suggested that low participation rates and high attrition rates in BPIs may, in part, be a consequence of the lengthy nature of treatment, and the associated burden placed on families [95]. Families have indicated a preference for briefer programs [96], and those who have participated in brief programs have reported high levels of satisfaction [97]. Further, the same treatment outcomes have been obtained in briefer, relative to longer, interventions [95]. Holding Hands thus endeavoured to provide a brief, yet effective, intervention for families.

On the basis of the review of evidence above, four central hypotheses were drawn in the present study. It was hypothesised that toddlers would demonstrate statistically significant improvement in emotional and behavioural functioning from pre- to post-treatment (as indicated on parent self-report measures). It was hypothesised that these gains would be maintained at three-month follow-ups. It was hypothesised that parent self-reported levels of stress, depression, and anxiety would demonstrate statistically significant improvement from baseline to post-treatment. Finally, it was hypothesised that these gains would be maintained at three-month follow-up.

## 2. Method

### 2.1. Participants and Screening

A total of 31 families with a child aged 12 to 48 months had completed the study by the time this evaluation was written. All families who were self-referred, or referred externally, to the University of Technology Sydney (UTS) Family Child Behaviour Clinic between August 2016 and September 2017, and who met inclusion criteria, were offered to participate in the study. A standardized telephone interview, containing a brief developmental-, social-, and risk-assessment determined eligibility for inclusion into the program. To be included, the target child must have been aged between 12 and 48 months (inclusive), have clinical or sub-clinical cut offs at intake on the Affective Problems, Anxiety Problems, and/or Oppositional Defiant Problems subscales of the Child Behaviour Checklist (CBCL), for ages 1½ to 5 [98]. Parents must have been able to attend weekly 90-minute sessions. The exclusion criteria, included the presence of Autism Spectrum Disorder, intellectual disability or other pervasive developmental features, parent report of significant marital/relationship difficulties, unmanaged substance use or reportable/reported child protection concerns.

**Figure 1:**
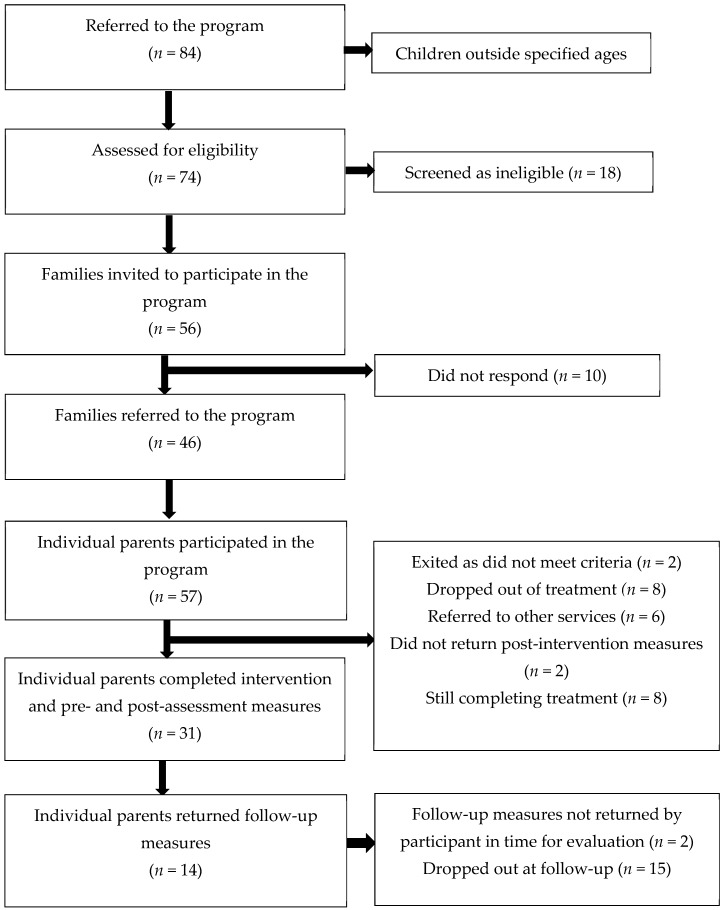
Flowchart for recruitment through screening, pre-assessment, post-assessment, and 3 month follow-up.

Figure 1 presents a flow chart for recruitment to the study. Of the 84 families who were self- or externally-referred to the program, 10 families were immediately referred to other services (for example, due to the age of the child or parent unable to attend weekly appointments), and 74 families were assessed for eligibility via a brief telephone interview. Of those who completed a screening interview, 56 (75%) families were eligible and were invited to participate in the study, and 46 (62%) attended the program. These families were made up of 57 individual parents, with 31 (54%) completing treatment and returning pre- and post-intervention measures. Only 14 (45%) parents completed measures at follow-up, as 15 (48%) had dropped out by this time (i.e., did not respond to request to return measures), and two (7%) parents had not returned their questionnaires by the time this evaluation was written.

The socio-demographic characteristics of the sample are summarized in Table 1. 

### 2.2. Design

A repeated-measures design was used, in which all participants received the Holding Hands program for between six and eight weeks. The independent variable was time (at two levels, including from pre- to post-treatment, and from post-treatment to follow-up) and the outcome variables were four subscales of the CBCL 1½ to 5 (Affective Problems, Anxiety Problems, Attention Deficit Hyperactivity Problems, and Oppositional Defiant Problems [98], three subscales of the Depression, Anxiety, and Stress Scales, Short Version (DASS-21) [99], and three subscales of the Parental Stress Index, Short Form (Parental Distress, Parent-Child Dysfunctional Interaction and Difficult Child) (PSI-SF) [100].

### 2.3. Materials

An observation room separated from an adjacent room by a one-way mirror, in addition to a Bluetooth hearing aid-device, was used to allow clinicians to discreetly observe families and coach them in their interactions. The psychometric measures used were as follows:

#### 2.3.1. The Child Behaviour Checklist for Ages 1½ to 5 

The Child Behaviour Checklist (CBCL) for ages 1½ to 5 [98], was used as a parent self-report measure of child emotional and behavioral functioning. Parents rated the extent to which each item was true of their child on 99, 3-point Likert Scale items. The measure yields a Total Problems score, an Externalising score, and an Internalising score. Items may be scored according to syndrome subscale scores, which represent common problems in children, including Emotionally Reactive, Anxious/Depressed, Somatic Complaints, Withdrawn, Sleep Problems, Attention Problems, and Aggressive Behaviour. Items may alternatively be scored according to the DSM-Oriented Scales, including Oppositional Defiant Problems, Anxiety Problems, Affective Problems, Attention Deficit Hyperactivity Problems, and Pervasive Developmental Problem. Scores falling in the 93rd to 97th percentile are considered borderline, and those above the 97th percentile are in the clinical range [98]. The CBCL 1 ½ to 5 has demonstrated strong psychometric properties [98].

#### 2.3.2. The Parental Stress Index, Short Form 

The Parental Stress Index-Short Form (PSI-SF) [100] was used as a parental self-report measure of stress in the context of parenting. Parents rated the extent to which each of 36 items applied to them on a 5-point Likert scale. Items are summed to yield a Total Stress score and three subscale scores, Parental Distress (distress resulting from his/her role as a parent), Parent-Child Dysfunctional Interaction (dissatisfaction with parent-child interactions), and Difficult Child (how parents perceive their children’s self-regulatory abilities). Higher scores on the PSI-SF are indicative of greater dysfunction, with Total Stress scores in the 90th percentile or above representing clinically significant parenting stress [100]. The PSI-SF has demonstrated strong psychometric properties [100,101,102].

#### 2.3.3. The Depression, Anxiety, and Stress Scales, Short Version

The Depression, Anxiety, and Stress Scales, Short Version (DASS-21); [99] was used as a self-report measure of parental psychological functioning. This 21-item scale is comprised of three subscales, relating to Depression (symptoms associated with dysphoric mood such as anhedonia, hopelessness, and low self-esteem), Anxiety (symptoms relating to anxious affect, such as shakiness), and Stress (general tension, irritability). Higher scores are indicative of greater psychopathology, with cut-off scores for each subscale indicative of mild, moderate, severe, and extremely severe symptoms. The DASS-21 has demonstrated sound psychometric properties [103,104]. 

### 2.4. Ethical Approval

Ethics approval was obtained from the University of Technology Sydney Human Research Ethics Committee (approval number: 2015000482-28), and the trail was registered under the Australian New Zealand Clinical Trials Registry (registration number: 12616001047482). Families were allocated to one of three clinicians (clinical psychologist or one of two provisional psychologists), and provided written informed consent prior to participation. Treatment was delivered consistently with the Holding Hands program manual, which emphasised that its content should be tailored to meet the individual needs and abilities of families. A total of six to eight 90-minute sessions were offered to families, depending on family need and mastery of skills. Parents completed the CBCL 1½ to 5, PSI-SF, and DASS-21 prior to attending their first session, after the last treatment session, and at 3-months following the completion of treatment. Follow-up calls, and referrals to the appropriate additional services, were provided where indicated. 

## 3. Results

Prior to conducting analyses, data was cleaned and all variables were assessed to determine they satisfied the assumptions of analyses of variance. Of the 31 parent participants engaged at pre-treatment, no drop-out was evident at post-treatment, although only 14 participants returned any follow-up data. Therefore, while the treatment analyses appear relatively robust, the treatment maintenance data should be treated with caution. The general trends, evident in means, presented in Table 2 below, suggest clear decreases in levels of each dependent variable from pre- to post-treatment, with mean scores remaining relatively stable between post-treatment and follow-up.

One-way repeated measures Multivariate Analysis of Variance (MANOVA), with a statistical significance set at *p* < 0.05, was conducted to examine a statistical significance of change in all dependant measures from pre- to post-treatment, and secondly from post-treatment to follow-up. As Mauchly’s Test of Sphericity indicated that the assumption of sphericity had been violated for all analyses (*p* < 0.001), Greenhouse-Geisser univariate analyses were used following significant overall tests, with a Bonferroni adjustment for multiple comparisons. The clinical significance of change from intake to post-intervention was additionally investigated with regards to emotional and behavioural functioning.

The inter-correlations of pre-intervention scores on the CBCL 1½ to 5, DASS-21, and PSI-SF are presented in Table 3 below. Scores on the CBCL 1½ to 5 Affective Problems subscale, were significantly positively correlated with the Oppositional Defiant Problems subscale and the Attention Deficit Hyperactivity Problems subscale, as well as with the DASS-21 Stress subscale, and the PSI-SF Difficult Child subscale. The PSI-SF Difficult Child subscale was also significantly positively correlated with the Oppositional Defiant Problems subscale and the Attention Deficit Hyperactivity Problems subscale of the CBCL 1½ to 5, as well as the DASS-21 Depression subscale.

### 3.1. Treatment Effects

To assess child emotional and behavioural treatment effects post-intervention, a one-way repeated measures MANOVA was undertaken to compare CBCL1 ½ to 5 scores at pre- and post- treatment. Results indicated significant within-subject differences in CBCL 1½ to 5 scores across these time points, F (4,22) = 13.396, *p* < 0.001, partial η^2^ = 0.709. Follow up Greenhouse-Geisser univariate analyses, with a Bonferroni adjustment, indicated significant decrease from pre- to post-program on all subscales of the CBCL 1½ to 5, including Affective Problems, F (1,25) = 32.655, *p* = 0.005, partial η^2^ = 0.566, Anxiety Problems, F (1,25) = 22.953, *p* < 0.001, η^2^ = 0.479, Attention Deficit Hyperactivity Problems, F (1,25) = 33.540, *p* < 0.001, partial η^2^ = 0.573, and Oppositional Defiant Problems, F (1,25) = 30.235, *p* < 0.001, partial η^2^ = 0.547. Partial eta squared values indicate that the change in each measure from pre- to post-treatment was relatively large [105].

To assess the treatment effects post-intervention on parental depression, anxiety, and stress, a one-way repeated measures MANOVA was undertaken to compare DASS-21 scores at pre- and post-treatment. Results indicated significant within-subjects differences in DASS-21 scores across Time, F (3,20) = 7.068, *p* < 0.001, partial η^2^ = 0.515. Follow-up Greenhouse-Geisser univariate analyses with Bonferroni adjustments indicated a statistically significant decrease in scores from pre- to post-treatment on all subscales, including Depression, F (1,22) = 9.731, *p* < 0.001, partial η^2^ = 0.307, Anxiety, F (1,22) = 14.778, *p* < 0.001, partial η^2^ = 0.402, and Stress, F (1,22) = 14.715, *p* < 0.001, partial η^2^ = 0.401. Partial eta squared values indicate that the change in each measure from pre- to post-treatment was relatively large [105].

To assess the treatment effects post-intervention on parental distress, parent child interactions, and parent’s report of their child as difficult or lacking self-regulatory abilities, a one-way repeated measures MANOVA was undertaken to compare PSI-SF scores at pre- and post-treatment. The results indicated significant within-subject differences on PSI-SF scores across these time points, F (3, 20) = 14.022, *p* < 0.001, partial η^2^ = 0.678. Follow-up Greenhouse-Geisser univariate analyses with Bonferroni adjustments indicated a statistically significant decrease in scores from pre- to post- treatment on all subscales, including Parental Distress, F (1,22) = 20.431, *p* < 0.001, partial η^2^ = 0.482, Parent Child Difficult Interaction, F (1, 22) = 13.038, *p* = 0.002, partial η^2^ = 0.372, and Difficult Child, F (1,22) = 35.979, *p* < 0.001, partial η^2^ = 0.621. Partial eta squared values indicate that the change in each measure from pre- to post-treatment was relatively large [105].

### 3.2. Post Intervention Effects

To assess the maintenance of child emotional and behavioural treatment gains post intervention, a one-way repeated measures MANOVA was used to compare CBCL 1½ to 5 scores at post-intervention to three months follow-up. Results indicated statistically non-significant within-subject differences in CBCL 1 ½ to 5 scores across these time points, F (4,9) = 0.438, *p* > 0.05, partial η^2^ = 0.149. A one-way repeated measures MANOVA was additionally used to assess the maintenance of treatment effects post-intervention on parental depression, anxiety, and stress, by comparing DASS-21 scores at post-treatment to follow-up. Results indicated statistically non-significant within subjects differences in DASS-21 scores across Time, F (3,10) = 0.536, *p* > 0.05, partial η^2^ = 0.139. Finally, to assess the maintenance of treatment effects post-intervention on parental distress, parent-child interactions, and parent’s perception of their child as difficult, a one-way repeated measures MANOVA was undertaken to compare PSI-SF subscales at post-treatment to follow-up. Results indicated statistically non-significant within subjects differences in PSI-SF scores across these time points, F (3,8) = 0.657, *p* > 0.05, partial η^2^ = 0.198.

### 3.3. Clinical Significance of Change in Emotional and Behavioural Functioning

The clinical significance of improvements in child emotional and behavioural functioning from pre- to post-intervention was also examined. A treatment effect may be considered a clinically significant change if it satisfies two criteria [106]: The change is statistically reliable (i.e., the Reliable Change Index, or RCI [107], and that scores reduce below the clinical threshold (i.e., clinical significance). Clinical norms were obtained from the CBCL 1½ to 5 manual [98]. Of the 15 children who scored in the clinical range on the Oppositional Defiant Problems subscale at pre-assessment, 13 (80%) experienced both a statistically reliable and clinically significant reduction in symptoms at post-assessment, and one (7%) experienced a statistically reliable reduction only. In terms of emotional functioning, of the eight individuals who scored in the clinical range on the Affective Problems subscale at pre-assessment, six (75%) experienced both a statistically reliable and clinically significant improvements in symptoms at post-assessment. Although no individuals were in the clinical range on the Anxiety Problems subscale at pre-intervention and thus could not meet the clinical significance, three individuals notably experienced a statistically reliable reduction in symptoms at post-intervention.

## 4. Discussion

As predicted, a statistically significant decrease in child emotional and behavioural problems, as reported by parents on the subscales of the CBCL 1½ to 5, was found from pre- to post-treatment and these gains were demonstrated to be maintained at follow-up. It is noteworthy that these changes were demonstrated to represent clinically significant change as well, suggesting that when applied in broader clinical settings, Holding Hands may be expected to return both statistically and clinically significant change. These findings are consistent with previous work demonstrating that changing parent-child interactional patterns, and the parental response to child behaviour, are effective in improving early emotional and behavioural problems [63,67,108]. The findings of the present study, however, also expand on previous work in important ways. 

Our findings may be taken to indicate that a treatment focus on the effective and contingent reinforcement of desirable behaviours, in the absence of employing exclusionary strategies in response to undesirable behaviours, is sufficient in creating meaningful change in toddlers. Holding Hands utilized four specific forms of contingent reinforcement in response to child behaviour that parents wanted to see more of: It asked parents to provide verbal, facial, physical and empathetic type responses to desirable child behaviour. Similarly, Holding Hands utilized two strategies in response to behaviour that parents wanted to see less of: These were a reduction in attention-based responding to that behaviour and an increase in parental calmness and non-reactivity in the face of behaviour they wanted to see less of. These two processes may represent a single mechanism or similar mechanisms of change. However, little research has been undertaken on the subject of increasing parental calmness and emotion-regulation in the context of parenting [41]. Improvements, particularly with regards to child emotional functioning, may result from the modelling or social learning influences of consistent parental emotion regulation [46,47], in addition to enhancing the parent-child relationship and increasing the child’s perceived mastery and control of their environment [17,51,53,56]. This may be facilitated in treatment by providing a platform of child-led play, and generalizing gains, from child-led play to broader parent-child interactions and the parent-child relationship. In Holding Hands, child-led play is called Kid-Time and children are encouraged to make autonomous decisions, while being reinforced in desirable behaviour by their parents, through contingent reinforcement on desirable behaviour, parental failure to respond to behaviour they want to see less of, demonstrable parental emotion regulation, and the removal of coercive or disproportionate discipline.

As hypothesised, parental depression, anxiety, and stress significantly decreased from pre- to post-treatment (as indicated on the DASS-21), and treatment gains were maintained at follow-up. From pre- to post-treatment, parents also reported significantly less parenting distress, more satisfaction regarding interactions with their child, and perceived their child as less difficult (as indicated on the PSI-SF), and this was maintained at follow-up. This is consistent with findings from previous BPIs [74,75], although the causal mechanisms of change are unknown. Facilitating such improvement is crucial given the previously documented relationship between increased parenting stress and undesirable child behaviour [35]. These findings may be attributed to a range of influences and, potentially, their interaction. We suggest that the delivery of effective parental emotion regulation skills may be strongly related to two additional outcomes, each of which bear a reciprocal relation to each other. Improvements in parental emotion regulation would likely positively impact on child management practices, both from the perspective of reducing attention-based responding to behaviour parents want to see less of, and increasing parental calmness and non-reactivity in the face of that behaviour. Similarly, improvements in parental emotion regulation would likely also positively impact on parental distress (including parental affect and parental anxiety) [57]. We suggest that it is also important to note the already documented relations between child management and parental distress [35,41], and believe that these three components may, in interaction, contribute to substantial improvements in parent-child relationships [35,75,109]. 

## 5. Conclusions

Whilst the present study establishes that Holding Hands is potentially a beneficial program in improving child and parent well-being, significant potential limitations must be acknowledged with this program. The small sample size associated with our trial may have reduced the indices of statistical significance and the clinically significant change we reported and may, as a result, have biased our findings. Maintenance of treatment gains, in particular, should be interpreted with caution given that only 14 participants had returned follow-up measures. In addition, as no control group was used, we are unable to rule out statistical and clinically significant change occurring due to maturational or other processes. Finally, from our point of view, significant benefits may have been gained in assessing the effectiveness of individual treatment components through the use of comparison groups. This was precluded, however, as the program was delivered holistically. 

There is much potential for future research to explore enhancing parent and child outcomes following engagement in BPIs. Future larger scale randomised controlled trials might further investigate whether combining Direct Coaching and PMT modes of delivery, as was the case in Holding Hands, is more effective than using either mode alone. Similarly, while our research sought to extend the use of a component designed to enhance parental emotion regulation, future research must evaluate this potential in clinical trials. The potential to develop effective programs of preventative intervention for young children, by utilizing non-exclusionary components in the absence of exclusionary components in order to address undesirable behavior, warrants further study. Future studies may also expand on self-report assessment measures used in this context. Future studies may, for instance, include observational measures of behaviour [110,111], as well as reports from independent raters (e.g., pre-school teachers). Measures may also be administered throughout the treatment in order to provide specific information about information about points of change. No drop-out occurred from pre- to post- intervention, indicating satisfaction and engagement with the program and its duration, although future studies must further explore other ways that the duration of BPIs can be reduced, while maintaining treatment response at both post and follow-up. The use of brief, effective programs has significant implications for public and private services delivering family health care. 

Overall, and with the appropriate caution, the current study represents a successful and informative pilot of the Holding Hands program. It indicates that an intervention, which is intensive and briefer than other programs; combines two forms of treatment (Direct Coaching and PMT); incorporates an emotion regulation component for parents; and utilises contingent reinforcement of desirable behaviours without employing exclusionary strategies in response to undesirable behaviour, can be effective in improving the emotional and behavioural functioning of toddlers and the wellbeing of their parents. It is possible that a trajectory towards enduring mental health problems later in life was interrupted for the vast majority of children in this study. If future research indicates that inclusion of these treatment components optimises treatment outcomes for families and addresses the limitations of current interventions (such as that of high dropout rates and low participation rates) this will have large clinical implications for families. Given the relatively lower success and greater difficulty intervening later in life [18,19], the availability of effective preventative interventions is crucial to prevent early emotional and behavioural problems becoming entrenched and from impacting on functioning later in life [2,3].

## Figures and Tables

**Table 1:** Socio-demographic characteristics of the 31 Families who completed the program.

Sociodemographic Variables	Mean (SD), Range
Child age (months)	31.5 (9.51), 16 to 47
Parent age (years)	37.1 (4.84), 27 to 46
	*n* (%)
Parent gender male	14 (45)
Child gender male	18 (60)
Child’s cultural group	
Caucasian	21 (70)
South East Asian	3 (10)
Chinese	2 (7)
European	2 (7)
Indigenous Australian	1 (3)
Parent employment	
Full time	16 (53)
Part time	3 (10)
Full time parenting	11 (37)
Parent education	
Year 12 or less	1 (3)
TAFE college/trade certificate	1 (3)
University degree	28 (94)
Family income	
$100,000 to $150,000	10 (38)
$180,000 to $190,000	3 (13)
$200,000 to $250,000	4 (15)
$300,000 to $350,000	9 (33)
$700,000	1 (1)
Child attends childcare	
1 day	6 (20)
2 days	7 (23)
3 days	5 (18)
4 days	4 (13)
5 days	8 (27)
Previous treatment for presenting concerns	2 (7)
Previous diagnosis	1 (3)

**Table 2:** Change in the Child Behaviour Checklist (CBCL) 1½ to 5, DASS-21, and Parental Stress Index, Short Form (PSI-SF) scores from pre- to post-treatment and follow-up.

Measure	Pre-Intervention			Post-Intervention			Follow-Up		
	*n*	Mean	SD	*n*	Mean	SD	*n*	Mean	SD
CBCL									
Affective Problems	31	4.54	3.06	31	1.90 *	1.65	14	2.00 ns	1.88
Anxiety Problems	31	4.14	2.59	31	2.10 **	2.91	14	1.86 ns	1.66
OD Problems	31	6.3	3.08	31	3.00 **	1.96	14	2.86 ns	1.35
ADH Problems	31	6.25	2.88	31	3.66 **	2.24	14	3.29 ns	2.13
DASS-21									
Depression	31	3.21	2.72	31	2.22 **	2.82	14	2.07 ns	2.67
Anxiety	31	2.56	1.91	31	1.14 **	1.84	14	1.21 ns	2.15
Stress	31	7.26	2.41	31	5.30 **	2.58	14	4.71 ns	2.52
PSI-SF									
DC	31	27.85	6.96	31	16.78 **	6.85	14	15.93 ns	7.3
PCDI	31	12.69	5.35	31	9.04 *	4.8	14	8.86 ns	5.43
PD	31	18.89	5.62	31	12.78 **	6.77	12	10.58 ns	7.43

Note: Attention Deficit Hyperactivity (ADH), Oppositional Defiant (OD), Difficult Child (DC), Parent Child Dysfunctional Interaction (PCDI), Parental Distress (PD). * *p* < 0.05 from pre- to post- intervention, ** *p* < 0.001 from pre- to post- intervention. ns = Nonsignificant difference between post-intervention and follow-up scores.

**Table 3:** Summary of Intercorrelations of Pre-intervention Scores on the CBCL 1½ to 5, DASS-21, and PSI-SF.

Measure	1	2	3	4	5	6	7	8	9
1. CBCL AffP			.						
2. CBCL AnxP	0.321								
3. CBCL ODP	569 **	0.106							
4. CBCL ADHP	0.518 **	−0.211	0.681 **						
5. DASS-21 Dep	0.091	−0.11	−0.002	0.267					
6. DASS-21 Anx	0.212	−0.048	0.047	0.303	0.403 *				
7. DASS-21 Stress	0.404 **	0.035	0.266	0.694 **	0.02	0.269			
8. PSI-SF DC	0.498 **	0.33	0.641 **	0.521 **	0.408 *	0. 075	0.256		
9. PSI-SF PCDI	0.354	0.258	0.042	−0.078	0.047	0.179	0.017	0.405 *	
10. PSI-SF PD	−0.17	−0.04	−0.113	0.231	0.348	0.390 *	0.296	0.18	−0.124

Note: Affective Problems (AffP), Anxiety Problems (AnxP), Oppositional Defiant Problems (ODP), Attention Deficit Hyperactivity Problems (ADHP), Depression (Dep), Anxiety (Anx), Difficult Child (DC), Parent Child Dysfunctional Interaction (PCDI), Parental Distress (PD). * *p* < 0.05. ** *p* < 0.001.
